# Chimeric antigen receptor-modified T cell therapy in chronic lymphocytic leukemia

**DOI:** 10.1186/s13045-018-0676-3

**Published:** 2018-11-20

**Authors:** Yixin Zou, Wei Xu, Jianyong Li

**Affiliations:** 1 Department of Hematology, The First Affiliated Hospital of Nanjing Medical University, Jiangsu Province Hospital, Nanjing, 210029 China; 2 Key Laboratory of Hematology of Nanjing Medical University, Nanjing, 210029 China; 3 Collaborative Innovation Center for Cancer Personalized Medicine, Nanjing, 210029 China

**Keywords:** Chimeric antigen receptor, Chronic lymphocytic leukemia, Immunotherapy, T cell, Toxicity

## Abstract

Chronic lymphocytic leukemia (CLL), a common type of B cell chronic lymphoproliferative disorder in adults, has witnessed enormous development in its treatment in recent years. New drugs such as ibrutinib, idelalisib, and venetoclax have achieved great success in treating relapsed and refractory (R/R) CLL. In addition, with the development of immunotherapy, chimeric antigen receptor-engineered T cells (CAR-T) therapy, a novel adoptive immune treatment, has also become more and more important in treating R/R CLL. It combines the advantages of T cells and B cells via ex vivo gene transfer technology and is able to bind targets recognized by specific antibodies without antigen presentation, thus breaking the restriction of major histocompatibility complex. So far, there have been lots of studies exploring the application of CAR-T therapy in CLL. In this review, we describe the structure of chimeric antigen receptor, the preclinical, and clinical results of CAR-T therapy against CLL, along with its adverse events and advances in efficacy.

## Background

Chronic lymphocytic leukemia (CLL) is a common type of B cell chronic lymphoproliferative disorder with varied treatment options as a result of its heterogeneity. It mainly affects the elderly, and the 5-year survival rate for CLL patients is 79.2% [1–3]. To date, it is still an incurable disease and a lot of patients, especially those with *tumor protein 53* (*TP53*) disruption, will relapse rapidly after first-line immunochemotherapy-based treatment. Therefore, new weapons are needed. New drugs such as ibrutinib and venetoclax have achieved great success even in relapsed and refractory (R/R) CLL. Besides, the great potential of immunotherapy in CLL also has already aroused worldwide interest.

Chimeric antigen receptor-engineered T cells (CAR-T) therapy, a novel adoptive immune treatment initially invented in 1989, is now attracting more and more attention because of its promising effects even for highly refractory hematological malignancies. Through gene transfer technology, T cells can express chimeric antigen receptor (CAR) targeting specific antigens. The advantage of CAR-T cells over conventional autologous T cells is that by equipping T cells with CAR, it can identify antigens via major histocompatibility complex independent recognition. Preclinical and clinical results show that CAR-T therapy is a feasible way to treat R/R CLL. By summarizing the recent advances, we hope to introduce in detail the application of CAR-T therapy in CLL and provide a new perspective for R/R CLL treatment.

### Structure of chimeric antigen receptor

CAR comprises four important elements, including an extracellular targeting domain, a hinge or spacer, a transmembrane domain and an intracellular signaling domain [4, 5]. Extracellular targeting domains are usually single chain variable fragments (scFv) consisting of heavy chain variable regions and light chain variable regions of immunoglobulin (Ig) [6]. A spacer/hinge, which often derives from human IgG1, IgG4, and CD8, is designed to connect scFv with transmembrane domain [7–9]. The length of this structure alters CAR activation and scFv flexibility by determining the distance between scFv and tumor antigen. A transmembrane motif takes auxiliary responsibility to assist scFv and intracellular signaling domain, and CD3ζ/CD28 are common molecules in it. Intracellular signaling domain is involved in CAR-T cells activation and proliferation. It is comprised of CD3ζ or FcεRIγ and co-stimulatory signaling domains such as CD28 or CD137 [10, 11]. Up to now, there are at least four generations of CAR-T cells, and the second generation is most commonly used. First generation has a single signaling molecule, and CD3ζ is most commonly used. Second generation can generate double signals via CD3ζ and a co-stimulatory domain [12, 13], and third generation has two co-stimulatory molecules. Fourth generation consists of multiple types, including TRUCK T cells [14] and CAR-T with suicide genes [15, 16]. Currently, most scFvs are murine-derived which may lead to clearance of CAR-T cells due to immunogenicity so that weakening the efficacy of the treatment. To solve this problem, CAR-T with human-derived scFv is developed and is superior to that with murine-derived scFv [17]. In addition, a new idea “universal CAR-T cells,” which are derived from healthy donors, could overcome the problems of dysfunction of T cells in patients and make “off-the-shelf” come true by editing genes of T cell receptor and human leukocyte antigen [18, 19]. CAR-T cells can be directed against tumor with certain antigen. However, under most circumstances, normal cells and tumor cells share some important antigens. In consideration of safety, researchers add programmed cell death protein 1 (PD-1) and cytotoxic T-lymphocyte-associated protein 4 (CTLA-4)-based inhibitory chimeric antigen receptors (iCAR) that can bind ligands on common cells to CAR-T cells. On this occasion, if these CAR-T cells come across normal cells, they will fail to activate [20]. By contrast, some investigators construct new CAR-T cells carrying chimeric co-stimulatory receptors (CCR) recognizing antigens existed in cancer cells so that they can only be activated when binding to two antigens expressed on tumor cells at the same time [21, 22]. Target antigen loss is a common reason for the failure of CAR-T therapy, but losing two antigen at the same time is rare. As a result, CD19-CD20 bispecific CAR-T cells are designed and showed their efficacy in killing CD19-negative CLL cells [23]. In addition, switchable dual-receptor CAR-engineered T cells were designed in order to make CAR-T cells controllable in the aspect of activation and toxicity [24].

### The application of CAR-T therapy in CLL: preclinical and clinical data

Choosing a satisfying target is a key step for CAR-T therapy. Chimeric antigen receptor can bind to a wide range of antigens that exist on the surface of cells, such as proteins, carbohydrate, ganglioside, proteoglycan, and heavily glycosylated protein [25–27]. Scientists hope to find a specific antigen only expressed on tumor cells, but this type of antigen has been difficult to come across. Other principles of a good target are that antigens are highly expressed on tumor cells and are the key elements to their function, while on normal cells their expression is weak or negative [28]. In 2009, Kochenderfer and his colleagues [29] had validated that CAR-T cells can specifically kill primary CLL cells. To date, a lot of potential antigens, such as CD19 and CD23, have been studied in preclinical and clinical research studies of CLL. Clinical trials of CAR-T therapy for patients with CLL are shown in Table 1.

**Table 1:** Open clinical trials of CAR-T-cell therapy for patients with CLL

ClinicalTrials ID	Trial title/description	Target
NCT02640209	Pilot Trial Of Autologous T Cells Engineered To Express Anti-CD19 Chimeric Antigen Receptor (CART19)In Combination With Ibrutinib In Patients With Relapsed Or Refractory CD19+ Chronic Lymphocytic Leukemia (CLL)Or Small Lymphocytic Lymphoma (SLL)	CD19
NCT02644655	Immunotherapy Using Autologous T Cell-Engineered With CD19-specific Chimeric Antigen Receptor for the Treatment of Recurrent /Refractory B Cell Leukemia	CD19
NCT02456350	Anti-CD19 Chimeric Antigen Receptor (CAR)-Transduced T Cell Therapy for Patients With B Cell Malignancies	CD19
NCT03076437	Anti-CD19 Chimeric Antigen Receptor (CAR)-Transduced T Cell Therapy for Patients With B Cell Malignancies	CD19
NCT01865617	Laboratory Treated T Cells in Treating Patients With Relapsed or Refractory Chronic Lymphocytic Leukemia, Non-Hodgkin Lymphoma, or Acute Lymphoblastic Leukemia	CD19
NCT02933775	CD19-redirected Autologous Cells (CAR-CD19 T Cells)	CD19
NCT02672501	A Study to Assess CD19-targeted Immunotherapy T Cells in Patients With Relapsed or Refractory CD19+ B Cell Leukemia	CD19
NCT01853631	Activated T-Cells Expressing 2nd or 3rd Generation CD19-Specific CAR, Advanced B-Cell NHL, ALL, and CLL (SAGAN)	CD19
NCT03110640	Anti-CD19 CAR T Infusion Combined With Allogeneic Stem Cell Transplantation for B-cell Leukemia/Lymphoma	CD19
NCT01747486	CD19 Redirected Autologous T Cells	CD19
NCT03085173	A Trial of “Armored” CAR T Cells Targeting CD19 For Patients With Relapsed CD19+ Hematologic Malignancies	CD19
NCT02685670	Competitive Transfer of #CD19-TCRz-CD28 and #CD19-TCRz-CD137 CAR-T Cells for B-cell Leukemia/Lymphoma	CD19
NCT02782351	Humanized CAR-T Therapy for Treatment of B Cell Malignancy	CD19
NCT00881920	Kappa-CD28 T Lymphocytes, Chronic Lymphocytic Leukemia, B-cell Lymphoma or Multiple Myeloma, CHARKALL	Kappa
NCT02050347	Activated T Lymphocytes Expressing CARs, Relapsed CD19+ Malignancies Post-Allo HSCT(CARPASCIO)	CD19
NCT02963038	CAR T Cells for Refractory B Cell Malignancy	CD19
NCT03068416	CD19-targeting, 3rd Generation CAR T Cells for Refractory B Cells Malignancy	CD19
NCT03166878	A Study Evaluating UCART019 in Patients With Relapsed or Refractory CD19+ Leukemia and Lymphoma	CD19
NCT03448393	CD19/CD22 Chimeric Antigen Receptor (CAR) T Cells in Children and Young Adults With Recurrent or Refractory CD19/CD22-expressing B Cell Malignancies	CD19/CD22
NCT03191773	A Study of Anti-CD19 CAR-T Cell Immunotherapy for Refractory /Relapsed B Cell Malignancies	CD19
NCT02132624	CD19-targeting 3rd Generation CAR T Cells for Refractory B Cell Malignancy - a Phase I/IIa Trial	CD19
NCT03277729	A Phase I/II Study to Evaluate the Safety of Cellular Immunotherapy Using Autologous T Cells Engineered to Express a CD20-Specific Chimeric Antigen Receptor for Patients With Relapsed or Refractory B Cell Non-Hodgkin Lymphomas	CD20
NCT02851589	Study Evaluating the Efficacy and Safety of PCAR-019 in CD19 Positive Relapsed or Refractory Leukemia and Lymphoma	CD19
NCT02819583	CAR-T Cell Immunotherapy in CD19 Positive Relapsed or Refractory Leukemia and Lymphoma	CD19
NCT03302403	Clinical Study of Redirected Autologous T Cells With a Chimeric Antigen Receptor in Patients With Malignant Tumors	CD19
NCT02706392	Genetically Modified T-Cell Therapy in Treating Patients With Advanced ROR1+ Malignancies	ROR1
NCT01626495	Phase I/IIA Study of CART19 Cells for Patients With Chemotherapy Resistant or Refractory CD19+ Leukemia and Lymphoma	CD19
NCT01475058	CD19 CAR T Cells for B Cell Malignancies After Allogeneic Transplant	CD19
NCT03331198	Study Evaluating Safety and Efficacy of JCAR017 in Subjects With Relapsed or Refractory CLL or SLL (TRANSCEND-CLL-004)	CD19
NCT01593696	Anti-CD19 White Blood Cells for Children and Young Adults With B Cell Leukemia or Lymphoma	CD19
NCT03436771	Long-term Follow-up Study for Patients Previously Treated With a Juno CAR T-Cell Product	CD19
NCT01864889	Treatment of Relapsed and/or Chemotherapy Refractory B-cell Malignancy by CART19	CD19
NCT03050190	A Phase I/II Multiple Center Trial of 4SCAR19 Cells in the Treatment of Relapsed and Refractory B Cell Malignancies	CD19

### CD19

CD19, a B cell surface protein, is expressed on almost all malignant B cells, normal B cells at different stages, and follicular dendritic cells, but hardly on other cells [30, 31]. Moreover, it is vital for B cell activation. The first clinical trial for CLL was launched in 2011, evaluating the efficacy of autologous anti-CD19 CAR-T cells (CD28 as the co-stimulatory molecule) in eight R/R patients. The first four patients did not receive cyclophosphamide while the next four patients did. Patients with and without cyclophosphamide preconditioning were infused with (1.2–3.0) × 10^7^ CAR-T cells/kg and (0.4–1.0) × 10^7^ CAR-T cells/kg, respectively. Eventually, patients without cyclophosphamide-conditioning had no response to CAR-T therapy and died from disease progression, while for the four patients treated with cyclophosphamide, three exhibited stable disease (SD), and one remained progressive (PD) [32]. The toxicities mainly included grades 1–2 fever, rigors, chills, and grade 3 febrile neutropenia. One patient taking 3.2 × 10^9^ CAR-T cells without cyclophosphamide suffered from grade 5 hypotension and renal failure. In other two studies, totally eight R/R CLL patients got cyclophosphamide and fludarabine followed by (0.1–2.8) × 10^7^ CAR-T cells/kg. Eventually, four patients achieved complete remission (CR), three partial remission (PR), and one SD [33, 34]. Grades 3 and 4 adverse events usually occurred in 8 days after CAR-T cells infusion, including hypotension, fever, acute renal failure, hypoxemia, hyperbilirubinemia, capillary leak syndrome, obtundation, liver enzymes/creatinine/electrolyte abnormalities, upper extremity thrombosis, and urinary tract infection. Besides, because of the elimination of normal B cells, some patients developed hypogammaglobulinemia. These results showed that CAR-T therapy did have the potential to control the disease resistant to chemotherapy. Compared with patients without previous lymphodepleting therapies, wonderful capacity of proliferation and persistence of CAR-T cells were seen in preconditioning patients because preconditioning might enhance the proliferation and persistence of CAR-T cells by reducing tumor burden, releasing tumor antigens, inducing inflammation, and eliminating immunosuppressive cells such as regulatory T cell and myeloid-derived suppressor cells [35–37]. Additionally, CAR-T cells would be eliminated because of murine single chain variable fragment. Therefore, preconditioning was needed to suppress autoimmunity in order to slow down the clearance of CAR-T cells [17]. Furthermore, these studies revealed that, in spite of receiving lower number of CAR-T cells, patients who got cyclophosphamide and fludarabine had higher overall response rate than those with cyclophosphamide only (87.5% vs 25.0%), which illustrated that using fludarabine and cyclophosphamide for preconditioning was superior to cyclophosphamide alone.

CD137, also known as 4-1BB, affects the persistence of CAR-T cells. Previous reports demonstrated that CAR-T coupled with CD28 could kill tumor cells effectively in the first 7 days, and CAR-T equipped with CD137 persisted longer [38, 39]. A comparison of CAR-T cells’ signaling domain based on published data was presented in Table 2. So far, there have been three published articles assessing the values of anti-CD19-CD137-CD3ζ CAR-T cells transfected with lentivirus [10, 40, 41]. A total of 14 CLL cases, including 6 *TP53* deficient patients, were infused with (0.14–11) × 10^8^ CAR-T cells after chemotherapy conditioning (six with bendamustine, three with fludarabine/cyclophosphamide, and five with pentostatin/cyclophosphamide). Eventually, four patients achieved CR and four PR. Totally nine patients suffered from grades 1–4 cytokine release syndrome (CRS), and the median occurrence day was 7. Tocilizumab or glucocorticoid was used in five patients, and four patients were admitted into the intensive care unit (ICU) because of hypotension and hypoxemia. In addition, neurotoxicity was seen in five patients, and almost all patients whose CAR-T treatment was effective had B cell aplasia and hypogammaglobulinemia. CAR copies could be detected after 1 year in patients with CR. Therefore, CAR-T cells coupled with CD137 transfected with lentivirus also showed beneficial and persistent effects on R/R CLL, similar to those with CD28.

**Table 2:** The outcomes of CAR-T therapy with different costimulatory molecules for CLL patients in published trials

Signal	Target	Study	Number	Preconditioning	Source	Cell dose	ORR and CRR
CD28	CD19	2011.Brentjens	8	None (3)	Autologous	Without preconditioning: 1.2–3.0 × 10^7^/kg	ORR 1/7 (14.3%)
							CRR 0/7 (0%)
				Cyclophosphamide (5)		With preconditioning: 0.4–1.0 × 10^7^/kg	One died before evaluation
		2012.Kochenderfer	4	Fludarabine + cyclophosphamide (4)	Autologous	0.3–2.8 × 10^7^/kg	ORR 3/4 (75.0%)
							CRR 1/4 (25.0%)
		2013.Cruz	4	None	Allogeneic	1.5–12 × 10^7^/m^2^	ORR 1/4 (25.0%)
							CRR 0/4 (0%)
		2015.Kochenderfer	4	Fludarabine + cyclophosphamide (4)	Autologous	1–4 × 10^6^/kg	ORR 4/4 (100%)
							CRR 3/4 (75.0%)
		2015.Kochenderfer 2016.Brudno	5	None	Allogeneic	0.4–3.1 × 10^6^/kg	ORR 2/5 (40.0%)
							CRR 1/5 (20.0%)
		2018.Geyer	8	Cyclophosphamide (8)	Autologous	3 × 10^6^/kg, 1 × 10^7^/kg 3 × 10^7^/kg	ORR 2/8 (25.0%)
							CRR 2/8 (25.0%)
	κ	2016. Ramos	2	None	Autologous	9.1 × 10^7^/m^2^	ORR 0/2 (0%)
						1.6 × 10^8^/m^2^	CRR 0/2 (0%)
CD137	CD19	2011.Kalos	14	Bendamustine (6)	Autologous	0.14–11 × 10^8^	ORR 8/14 (57.1%)
		2011.Porter		Fludarabine/cyclophosphamide (3)			CRR 4/14 (28.6%)
		2015.Porter		Pentostatin/cyclophosphamide (5)			
		2017.Turtle	24	Fludarabine + cyclophosphamide (21)	Autologous	2 × 10^5^/kg, 2 × 10^6^/kg	ORR 16/23 (69.6%)
				Fludarabine (2)			CRR 4/23 (17.4%)
				Cyclophosphamide (1)		2 × 10^7^/kg	One died before evaluation

*Abbreviations*: *ORR* overall response rate, *CRR* complete remission rate

The function of T cells is usually impaired, even exhausted in CLL patients, which may restrict the capacity of CAR-T cells. Accordingly, relevant studies using allogeneic retrovirally transduced anti-CD19-CD28ζ CAR-T cells were carried out in the past 5 years in order to explore whether using donor-derived T cells was a good approach to overcome this limitation. A total of nine R/R CLL subjects who relapsed after allogeneic hematopoietic stem-cell transplantation took part in clinical trials, and none of them received chemotherapy conditioning before infusing (1.5–12) × 10^7^/m^2^ or (0.4–3.1) × 10^6^/kg CAR-T cells. Consequently, one patient exhibited CR, two PR, two SD, and four PD. No graft-versus-host disease occurred after infusion, and common side effects were fever and hypotension. Tumor lysis syndrome was seen in one patient [42–44]. Lack of previous chemotherapy conditioning and low dosage of CAR-T cells may account for the relatively low response rate. However, donor-derived CAR-T therapy is still a promising approach for treating R/R CLL because of the excellent state of donor T cells and graft versus leukemia effects, and someday “off-the-shelf” may be possible [45].

In the era of novel drugs, ibrutinib, a Bruton’s tyrosine kinase (BTK) inhibitor, is the first choice for first-line and R/R therapy for CLL with 17p deletion or *TP53* mutation [46]. It remains unclear how to treat CLL patients after failure of ibrutinib. Turtle et al. [11] evaluated the feasibility of using CAR-T therapy for CLL patients who were refractory to ibrutinib. It was a dose escalation trial, and a total of 24 patients, most of whom had a complex karyotype or 17p deletion, received lymphodepleting conditioning followed by infusion of 2 × 10^5^, 2 × 10^6^, or 2 × 10^7^ CAR-T cells/kg. The overall response rate was 71% at 4 weeks. The percentage of patients who were absent of marrow disease detected by flow cytometry and absent of marrow malignant *immunoglobulin heavy chain* (*IGH*) clone detected by deep *IGH* sequencing was 88% and 58%, respectively. However, the incidence of CRS and neurotoxicity was 83% and 33%, respectively, which was higher than that in previous reports. The number of grades 1–2 CRS, grade 4 CRS, and grade 5 CRS were 18, 1, and 1, respectively. The number of grades 1–2, grade 3, and grade 5 neurotoxicity were 2, 5, and 1, respectively. Neurotoxicity was reversible, and it was always associated with CRS. In total, six patients needed tocilizumab or glucocorticoid for CRS, and two patients needed ICU treatment for neurotoxicity. Positron emission tomography-computed tomography (PET-CT) was useful for lymph node response evaluation in CAR-T therapy. Some CLL patients classified as PR by the International Workshop on Chronic Lymphocytic Leukemia (IWCLL) were restaged as CR after PET-CT scan due to no lesions with fluorodeoxyglucose uptake. Despite low infusion dose, the overall response rate acquired in ibrutinib-resistant patients were satisfactory comparing with results reported by Brentjens et al. [32] in 2011. In Brentjens et al. study, all patients had bulky lymphadenopathy, and did not receive preconditioning or only got cyclophosphamide. The mean CD4/CD8 ratio in cellular products was 10.5, which was much higher than 1, the ideal ratio for CAR-T therapy [47]. The persistence time and the ability of cytokine release of CAR-T cells were inferior to those in Turtle’s research, which might be due to the relatively low level of manufacturing technique of CAR-T cells in 2011. Besides, ibrutinib could improve T cell function in CLL patients, and eventually, enhance the efficacy of CAR-T therapy. In summary, CAR-T therapy is a good choice for patients after failure of ibrutinib. It is worth mentioning that control of adverse events and application of PET-CT are vital in this process.

Additionally, combining ibrutinib with anti-CD19 CAR-T was a promising idea to treat heavily pretreated CLL individuals or untreated CLL patients with *TP53* disruption. A total of 10 CLL patients were enrolled in a pilot trial and none of them achieved CR when treated with ibrutinib. They were lymphodepleted before CAR-T infusion and continued to take ibrutinib. At 3 months, 89% of evaluable patients achieved a minimal residual disease (MRD)-negative marrow CR. CRS was seen in nine patients, but tocilizumab was not required. Compared with single ibrutinib or CAR-T, combination therapy had a better outcome and controllable toxicity, and therefore, ibrutinib and CAR-T had a synergistic effect to treat high-risk CLL patients, especially those with *TP53* disruption [48].

Using CAR-T cells as a consolidative therapy was a novel concept presented by Geyer and his colleagues [49]. Totally eight patients who had residual CLL cells after pentostatin, cyclophosphamide, and rituximab treatment were enrolled in this clinical trial. All patients had poor prognostic factors such as unmutated *immunoglobulin heavy chain variable region* gene. Patients received low-dose chemotherapy with 600 mg/m^2^ cyclophosphamide, followed by infusion of 3 × 10^6^, 1 × 10^7^, or 3 × 10^7^ anti-CD19-CD28-CD3ζ CAR T cells/kg. Eventually, two patients achieved CR, and no severe CRS and neurotoxicity were seen after CAR-T cells infusion. Persistence time of CAR-T cells was relatively short in these CLL patients, with the longest time was 48 days after infusion.

### CD23

CD23 (FcepsilonRII), a low-affinity IgE receptor, is involved in IgE synthesis. The typical phenotype of CLL cells is CD5^+^CD23^+^, which makes CD23 a potential target for CLL therapy. The feasibility and safety of anti-CD23 CAR-T cells were tested in a recent study [50]. Notably, the results showed that in vitro, CAR-T cells could effectively lyse either CLL-like MEC-1 cells or primary CLL cells and release cytokines such as interferon-γ (IFN-γ) without serious toxicity for normal B cells. Additionally, anti-CD23 CAR-T cells can slow the proliferation of tumor cells in a CLL xenograft mouse model. Therefore, preclinical results showed that anti-CD23 CAR-T cells could eliminate CLL cells effectively in vitro and in vivo. Similar to the sequential infusion of anti-CD19 and anti-CD22 CAR-T cells in treating R/R acute lymphocytic leukemia (ALL) as well as B cell lymphoma, and combined infusion of CD19 and B cell mature antigen (BCMA)-specific CAR-T cells for R/R multiple myeloma, combining anti-CD19 with anti-CD23 CAR-T cells to treat R/R CLL might be a promising strategy as well.

### ROR1

Tyrosine kinase-like orphan receptor 1 (ROR1), also known as neurotrophic tyrosine kinase, receptor-related 1, is a member of the receptor tyrosine kinase-like orphan receptor family. ROR1 is highly expressed on CLL cells, but not on normal B cells, which makes ROR1 an ideal tumor-specific antigen for antitumor immunotherapy [51]. Preclinical data demonstrated that CAR-T cells can accurately recognize autologous ROR1-expressing tumor cells or cell lines and could serve as a powerful weapon to eliminate chemotherapy resistant CLL cells. However, it should be noted that on-target/off-tumor toxicity must be taken into consideration when using anti-ROR1 CAR-T because of its expression on other normal tissues, such as parathyroid, pancreatic islet cells, and gastrointestinal tract [52–55].

### κ light chain

CLL cells are originated from monoclonal B lymphocytes and restrictively express κ or λ light chain. Unlike CD19, CAR-T cells targeting κ or λ chain can only kill a fraction of normal B cells, which may alleviate the adverse effect of B cell aplasia. A phase 1 dose escalation clinical trial, in which two κ^+^ R/R CLL patients were involved, was designed to evaluate the role of anti-κ CAR-T cells in hematologic malignancies. Neither of them received lymphodepleting conditionings. One patient exhibited no response after infusion with 9.1 × 10^7^/m^2^ CAR-T cells once, and the other, who was infused with 1.6 × 10^8^/m^2^ CAR-T cells twice, eventually showed stable disease lasting 6 weeks. No serious adverse events were seen in these two patients. Low infusion dosage, low expression of κ on the surface of CLL cells, and no lymphodepleting conditionings may explain these unsatisfactory results and the feasibility of anti-κ CAR-T cells in CLL needs further validation [56].

### CD20

CD20 is a mature B cell antigen limitedly existing on the surface of CLL cells. Nowadays, many novel immunotherapy drugs targeting CD20, such as rituximab, ofatumumab, and GA101, can exert their function to kill CLL cells by antibody-dependent cell-mediated cytotoxicity (ADCC) or complement-dependent cytotoxicity. However, CLL cells may be gradually refractory to these drugs after a few days of therapy by downregulating the expression of CD20. A recent study showed that CLL cells, which were resistant to CD20 monoclonal antibodies, could be lysed by anti-CD20 CAR-T cells. Further study demonstrated that the threshold density of CD20 was nearly 200 molecules per cell for CAR-T lysis activity and several thousand for CAR-T cytokine release [57]. Therefore, if we can choose a target whose density on CLL cells is between the threshold of lysis activity and cytokine release, the incidence of CRS may greatly decrease.

### FcμR

Immunoglobulin M Fc receptor (FcμR), also known as the Fas apoptotic inhibitory molecule-3 or TOSO, is selectively expressed on CLL cells and limitedly expressed by normal B cells. Faitschuk et al. [58] confirmed that anti-FcμR CAR-T cells, derived from CLL patients with different disease status, could effectively kill CLL cells in vitro and in vivo by releasing a variety of factors, but not kill normal cells, thus avoiding off-target effect to a certain degree. Preclinical data reveals that FcμR is superior to previous target antigens such as CD19 and CD23 and has the potential to have a place in CLL immunotherapy.

### CD16 V158

All of the CAR-T cells mentioned previously directly kill tumor cells. It is known to all that natural killer cells, which have a CD16 V158 variant, can exert ADCC by binding to the Fc fragment of immunoglobulin. Based on it, Kudo et al. [59] designed a kind of CAR-T cells containing CD16 V158 scFv so that they could eliminate CLL cells effectively by the regulation of rituximab at a small count. Besides, rituximab combined with anti-CD16 V158 CAR-T cells showed a better capacity to kill CLL cells than rituximab only. In all, anti-CD16 V158 CAR-T cells can enhance the efficacy of rituximab and have the potential to be a universal approach in the future.

### Adverse events

CRS, B cell aplasia, neurotoxicity, and infection are common adverse events of CAR-T cell therapy in CLL [60–62]. When encountering target cells, CAR-T cells are activated in a short time and release many kinds of cytokines such as interleukin (IL)-6, IL-10, tumor necrosis factor (TNF)-α, and IFN-γ at high-levels. In addition, macrophages, monocytes, natural killer cells, and dendritic cells can also be involved in the inflammatory response by producing inflammatory factors under the stimulation of CAR-T cells, target cells, and inflammatory mediators [63]. Recent studies showed that monocytes/macrophages were the major sources of IL-1 and IL-6 during CRS [64, 65]. The severity of CRS is positively associated with high bone marrow tumor burden, thrombocytopenia before preconditioning, number of CAR-T cells, and so on [61]. The incidence of CRS is relatively high in CLL (CRS occurs in nearly 83% CLL patients), especially for patients who used ibrutinib previously, which may be due to the recovery of T cell exhaustion [11]. CRS can affect multiple organ systems, and clinical symptoms of CRS in CLL are various, ranging from fever and hypoxia to renal failure and even death [34]. IL-6 receptor antagonist tocilizumab and corticosteroids are two desirable regimens which are often used to attenuate symptoms when necessary [66]. Tocilizumab is used with the dose of 4 to 8 mg/kg (not to exceed 800 mg) and infused over 1 h. When CRS is refractory to tocilizumab, methylprednisolone can be used with the dose of 1–2 mg/kg every 12 h. In addition, dexamethasone can be used with the dose of 10 mg every 6 h. All these drugs are used according to different clinical symptoms [67]. Because CD19 is widely expressed on B cells, anti-CD19 CAR-T cells can both eliminate CLL cells and normal B cells, causing B cell aplasia, which is characterized by the deficiency of B cells and immunoglobulin [33]. When it occurs, extra-immunoglobulin infusion is a feasible solution in order to refrain from infection [12]. In the past 5 years, neurologic toxicity, whose mechanism remains unclear, has been reported in a few CLL patients and clinicians are gradually aware of this issue. The incidences of grades 1–3 neurotoxicity and grades 4–5 neurotoxicity in CLL are 29% and 4% respectively [62]. Typically, its symptoms include delirium, aphasia, seizure, etc. Time of first fever after the CAR-T cell infusion is negatively correlated with the severity of neurotoxicity. Luckily, most neurologic toxicity is reversible and can recover spontaneously without further treatment. At present, CAR-T-cell-therapy-associated toxicity 10-point neurological assessment score is recommended to test encephalopathy syndrome of patients. When neurologic toxicity is severe, high-dose corticosteroids are recommended to use. Additionally, anti-IL-6 therapy such as tocilizumab or siltuiximab can be used if neurologic toxicity is associated with concurrent CRS [68]. Peak level of CAR-T cells in blood is positively associated with efficacy and toxicity in CLL. When the number of peak CD8^+^ and CD4^+^ CAR-T cells approximately reach to 100/μl and 30/μl, respectively, the estimated probability of marrow MRD-negative CR, grade ≥ 2 CRS and grade ≥ 3 neurotoxicity are 95%, 60% and 30%, respectively [61]. Infection is a severe toxicity after CAR-T treatment because of hypogammaglobulinemia and neutropenia, sometimes even causing death. The incidence of grade ≥ 3 infection in CLL is 21%–25% according to previous reports and is significantly lower than ALL. Bacteria are the most common causes, and viruses as well as fungi are also involved in it [60]. Top-level antibiotics, antifungal agents, and antiviral agents are indispensable when infection occurs. According to the latest data of ALL, CLL, and lymphoma, among events with specific pathogens, all bacterial infections were opportunistic infections, including coagulase-negative *Staphylococcus aureus* (4/11), *Streptococcus* (2/11), *Enterococcus faecium* (1/11), *Escherichia coli* (1/11), *Acinetobacter ursingii* (1/11), *Stenotrophomonas maltophilia* (1/11), and *Capnocytophoga sputigena* (1/11). *Mycoplasma hominis* was also reported in one patient. Opportunistic infections of viruses included cytomegalovirus (1/13) and Epstein-Barr virus (2/13), and fungal infections included *candida* (4/5) and *Aspergillus ustus* (1/5). Prior anti-tumor treatment regimens ≥ 4, high dose of CAR-T cells, ANC < 500 cells/μl on the day of infection and high grade of CRS/neurotoxicity were high-risk factors of infection [60].

### Advances in efficacy

CAR-T cells can effectively eliminate CLL cells, however, not all R/R CLL patients can benefit from this treatment. Previous clinical trials revealed that the overall response rate was 53% [69], but only 26% of individuals could achieve sustained remission [70]. As a consequence, finding feasible biomarkers to predict efficacy and exploring valid strategies to improve functions of CAR-T cells are essential in order to take full advantage of this weapon.

Fraietta et al. [70] retrospectively analyzed 41 R/R CLL patients who received CAR-T cell therapy, and by comparing the different characteristics of effective group and invalid group, they found that efficacy of CAR-T cells was determined by intrinsic potency, memory-related gene expression, subpopulation, cytokines, and so on, while it was not associated with age, previous treatment, tumor burden, or *TP53* states. After infusion, the number of CAR-T cells in peripheral blood would immediately decline to a low level because of redistribution in peripheral blood, bone marrow, and other tissues, and then undergo rapid expansion. During the process of manufacture and infusion, CAR-T cells derived from CR patients showed superior capacity of expansion and persistence than nonresponding (NR) patients, which was reflected in the higher peak level and the longer half-life, but time to peak level was no different between two groups [69]. These demonstrated that high peak absolute number of CAR-T cells, favorable reactivity to target cells, and persistent existence were indispensable in guaranteeing efficacy. Transcriptomic profiles showed that CAR-T cells from CR patients were enriched in early memory-associated genes while T cells from NR patients showed upregulated expression of late memory, effector, apoptosis, or aerobic glycolysis-related genes. Regarding cell types, the frequency of CD8^+^ T memory stem cells (*T*_SCM_) and CD27^+^CD45RO^−^CD8^+^ T cells were higher in CR patients than in NR patients. The differentiation of T cells is from early memory to effector memory stage, and metabolism changes from anabolism to catabolism. The differences of transcriptomic profiles and cell types of CAR-T cells between CR and NR patients suggested that CAR-T cells from CR patients are in early stage of differentiation and have strong proliferative ability and long-term survival, while CAR-T cells from NR patients are in terminal stage and have reasonable but unsustainable effector function [71]. Additionally, infusing CD27^+^PD-1^−^CD8^+^ CAR-T cells is essential in order to achieve favorable efficacy because CD27^+^PD-1^−^CD8^+^ CAR-T cells had high expression of IL-6 receptor-β chain and when stimulated by IL-6, levels of phosphorylated signal transducer and activator of transcription 3 (STAT3) rose and CAR-T cells quickly expanded. No significant difference was seen in the subset of CD4^+^ T cells between CR and NR patients. Moreover, concentrations of serum IL-15 and IL-6 were higher in CR patients than in NR patients, which are useful for CAR-T cells’ proliferation.

Early immune deficiency is a hallmark of CLL patients, and the prominent feature is T-cell exhaustion. Under the stimulation of a large number of malignant B cells like chronic viral infections, T cells gradually lose vigor and vitality [72]. In addition, drugs like fludarabine can also have an immunosuppressive impact on T cells, especially for CD4^+^ T cells. Compared with healthy persons, the expression of exhaustion markers, such as PD-1, CD160, and CD244, is upregulated on the surface of T cells from CLL patients. Besides, CD8^+^ T cells also show impaired function of proliferation, cytotoxicity, and cytolysis, while the ability of cytokine release is normal [73]. Moreover, CAR-T cells derived from CD4^+^ naïve T cells (T_N_) cells of CLL patients show the inferior ability of expansion and express a higher level of PD-1 than healthy individuals [72]. On this occasion, CAR-T cells derived from CLL patients are unable to expand and kill neoplasms normally. Consecutive ibrutinib treatment for more than 20 weeks can augment CAR-T cells’ ability in the aspects of proliferation, persistence, and cytotoxicity in vitro and in vivo, while also promote the reconstitution of cellular immune by reducing the expression of immunosuppressive molecule PD-1 on CD8^+^ T cells and CD200 on B-CLL cells, increasing the IFN-γ secretion of CD8^+^ T cells, inhibiting IL-2 inducible T-cell kinase in CD4^+^ T cells, driving CD4^+^ T cells to develop into T helper 1-type, and increasing T cell repertoire diversity [74, 75]. Results of combining CAR-T cells with ibrutinib in CLL xenograft models were consistent with those in the clinical trials [48].

According to the different differentiation states, cell surface molecules and functions, T cells are mainly divided into four subgroups: *T*_N_, effector memory T cells (*T*_EM_), central memory T cells (*T*_CM_), and T effector cells (*T*_E_) [71]. Previous studies demonstrated that in CLL treated with either CD4^+^ or CD8^+^ CAR-T cells, T_N_, and T_CM_ were superior to *T*_EM_ and *T*_E_ in anti-tumor activity, and they showed great abilities of proliferation and cytotoxicity. Unfortunately, CAR-T cellular products of CLL patients always exhibit low *T*_N_/*T*_E_ ratio and thus can only exert limited function in subsequent therapy. IL-7/IL-15 can induce CAR-T cells to differentiate into *T*_N_, *T*_SCM_, while CAR-T cells stimulated by IL-2 are susceptible to become *T*_EM_ and regulatory T cells. Therefore, it is a good choice to use IL-7/IL-15 as stimulants in the process of manufacture [72]. In addition, cooperativity is seen when using *T*_N_ and *T*_CM_ at the same time, and a defined ratio of CD4/CD8 such as 1:1 lead to better outcomes [47, 76]. Unfortunately, the CD4/CD8 T cells ratio of heavily pretreated CLL patients is far from 1:1 and the subsets are mainly *T*_EM_ and *T*_E_. Therefore, reallocating CD4/CD8 ratio and choosing *T*_N_ and *T*_CM_ by sorting T cells according to different phenotype are desirable methods to enhance CAR-T efficacy in CLL.

*TET2* can control the proliferation of blood cells by regulating their formation. A recent study demonstrated that in a CLL patient who received CAR-T therapy, the structure of *TET2* was disrupted when CAR lentivirus inserted into it, and consequently, CAR-T cells could proliferate capriciously without the inhibition of *TET2*. In this condition, more than 90% of CAR-T cells in this patient’s body have the same T cell receptor beta repertoire, which means they are from the same cell. What is more, CD8^+^ CAR-T cells with disrupted *TET2* tend to differentiate into *T*_CM_, characterized as early memory, rather than *T*_E_, so that they can last for a long time, and patients can maintain CR status. However, these changes may result in uncontrollable proliferation of CAR-T cells, which could generate another form of leukemia and eventually do more harm than good to CLL patients. To solve this problem, adding suicide genes or CD20 antigen into *TET2* disrupted CAR-T cells should be taken into consideration in order to ensure the safety of patients [77].

## Conclusions

In the last 2 years, enormous progress was witnessed in the aspect of CAR-T therapy for CLL. However, no comprehensive review had been reported so far. This review summarizes the development of CAR-T therapy in CLL and is intended to raise physicians’ awareness of cellular therapy. Nowadays, CAR-T therapy, a promising adoptive T cell immunotherapy, has become more and more important in the treatment of CLL. As an incurable disease, CLL will relapse after a few years. For patients with adverse prognostic features, such as *TP53* disruption and unmutated immunoglobulin heavy chain variable region, their diseases will either relapse in a short time or can be resistant to traditional immunochemotherapy. Even in the era of novel drugs, drug resistance will occur in some patients. CAR-T therapy can effectively eradicate CLL cells in R/R patients, even for those resistant to ibrutinib. At present, CAR-T therapy and stem cell transplantation are recommended as ultimate weapons for R/R CLL patients [78]. In general, the overall response rate is 53% [69], but only 26% of individuals can achieve sustained remission [70]. Preconditioning is necessary before infusion of CAR-T cells, and adverse events are manageable with current infusion dose. Because of the heterogeneity of these clinical trials, no significant difference is seen between co-stimulatory molecule CD28 and CD137. In general, the ALL patients are younger than CLL patients. Besides, the microenvironment of CLL inhibits the activation and proliferation of T cells. These two reasons account for the inferior efficacy of CAR-T therapy for CLL than that for ALL [79, 80]. Certainly, many problems need to be solved in CAR-T therapy for CLL. For example, which antigen to choose, how to alleviate adverse events, and how to enhance efficacy. Furthermore, T cell senescence and CAR-T cell exhaustion receive more and more attention [81]. To some extent, these problems limit the application of CAR-T therapy in CLL. In the future, combination with new drugs such as ibrutinib, venetoclax, and idelalisib, and sequential infusion of CAR-T cells targeting multiple antigens like CD19, CD23, and FcμR may be the trend of CAR-T therapy for CLL. Furthermore, researchers should try their best to formulate suitable projects to treat CLL patients with CAR-T therapy, so that eventually, patients can benefit from this weapon.

## Abbreviations

ADCC: Antibody-dependent cell-mediated cytotoxicity
ALL: Acute lymphocytic leukemia
BCMA: B cell mature antigen
BTK: Bruton’s tyrosine kinase
CAR: Chimeric antigen receptor
CAR-T: Chimeric antigen receptor-engineered T cells
CCR: Chimeric co-stimulatory receptor
CLL: Chronic lymphocytic leukemia
CR: Complete remission
CRS: Cytokine release syndrome
CTLA-4: Cytotoxic T-lymphocyte-associated protein 4
FcμR: Immunoglobulin M Fc receptor
iCAR: Inhibitory chimeric antigen receptor
ICU: Intensive care unit
IFN-γ: Interferon-γ
Ig: Immunoglobulin
IGH: Immunoglobulin heavy chain
IL: Interleukin
IWCLL: International Workshop on Chronic Lymphocytic Leukemia
MRD: Minimal residual disease
NR: Nonresponding
PD: Progressive disease
PD-1: Programmed cell death protein 1
PET-CT: Positron emission tomography-computed tomography
PR: Partial remission
R/R: Relapsed and refractory
ROR1: Tyrosine kinase-like orphan receptor 1
scFv: Single-chain antibody fragment
SD: Stable disease
STAT3: Signal transducer and activator of transcription 3
*T*_CM_: Central memory T cells
*T*_E_: T effector cells
*T*_EM_: Effector memory T cells
*T*_N_: Naïve T cells
TNF: Tumor necrosis factor
TP53: Tumor protein 53
*T*_SCM_: T memory stem cells
